# Methods and extractants to evaluate silicon availability for sugarcane

**DOI:** 10.1038/s41598-018-19240-1

**Published:** 2018-01-17

**Authors:** Carlos Alexandre Costa Crusciol, Dorival Pires de Arruda, Adalton Mazetti Fernandes, João Arthur Antonangelo, Luís Reynaldo Ferracciú Alleoni, Carlos Antonio Costa do Nascimento, Otávio Bagiotto Rossato, James Mabry McCray

**Affiliations:** 1 São Paulo State University (UNESP), College of Agricultural Sciences, Dep. of Crop Science, Lageado Experimental Farm, P.O. Box 237, 18610-307 Botucatu, São Paulo Brazil; 2 UNESP, Center of Tropical Tubers and Starches (CERAT), Lageado Experimental Farm, P.O. Box 237, 18610-307 Botucatu, São Paulo Brazil; 3 Oklahoma State University, Plant and Soil Sciences Department, Agricultural Hall, Stillwater, Oklahoma 74078-6028 USA; 4 University of São Paulo (USP), Escola Superior de Agricultura Luiz de Queiroz, Dep. of Soil Science, P.O. Box 09, 13418-900 Piracicaba, São Paulo Brazil; 5 Instituto Federal Catarinense, Campus Concórdia, Rodovia SC 283, Fragosos, 89703-720 Concordia, SC Brazil; 6 University of Florida, Everglades Research and Education Center, Belle Glade, Florida 33430-4702 USA

## Abstract

The correct evaluation of silicon (Si) availability in different soil types is critical in defining the amount of Si to be supplied to crops. This study was carried out to evaluate two methods and five chemical Si extractants in clayey, sandy-loam, and sandy soils cultivated with sugarcane (*Saccharum* spp. hybrids). Soluble Si was extracted using two extraction methods (conventional and microwave oven) and five Si extractants (CaCl_2_, deionized water, KCl, Na-acetate buffer (pH 4.0), and acetic acid). No single method and/or extractant adequately estimated the Si availability in the soils. Conventional extraction with KCl was no more effective than other methods in evaluating Si availability; however, it had less variation in estimating soluble Si between soils with different textural classes. In the clayey and sandy soils, the Na-acetate buffer (pH 4.0) and acetic acid were effective in evaluating the Si availability in the soil regardless of the extraction methods. The extraction with acetic acid using the microwave oven, however, overestimated the Si availability. In the sandy-loam soil, extraction with deionized water using the microwave oven method was more effective in estimating the Si availability in the soil than the other extraction methods.

## Introduction

In recent decades, the number of studies on silicon (Si) has increased substantially in many crops due to the beneficial effects of Si application on crop yields and plant resistance to biotic and abiotic stresses^1–7^. Silicon provides several benefits such as disease control, pest control, toxicities and freezing alleviations, water economy, and improving erectness and yield for many Si-accumulating and non-Si-accumulating plants, particularly sugarcane (*Saccharum* spp. hybrids), which absorbs more Si than any other mineral nutrient and accumulates approximately 380 kg ha^−1^ Si in a 12-month-old crop^8^.

The difficulty of accurately assessing the Si availability in the soil has been a limiting factor in the use of Si in agriculture and in the development of research related to this element^9–11^ because it is crucial to know the precise availability of Si in soil to adjust the recommendations of Si fertilization^10^. Si is present primarily in the soil in the following forms: i) soluble Si, present in soil solution; ii) Si adsorbed or precipitated with iron oxides (Fe_2_O_3_) and aluminum oxides (Al_2_O_3_); and iii) Si as a component of crystalline or amorphous silicate minerals^12–15^. In highly weathered humid tropical regions, Si is found preferentially in sesquioxides (oxides, oxyhydroxides, Fe hydroxides and Al hydroxides)^16–19^. The importance of Si cycling through the phytogenic Si pool, mainly composed by the phytoliths, has been currently emphasized^13,20,21^. Harvesting practices applied for sugarcane crop, without trash burning can significantly increase this pool^14,15^. Phytoliths are highly resistant to dissolution and may remain in soils for thousands of years^15^. For soil under sugarcane cultivation, we also must take into consideration the contribution of phytogenic soluble Si (Si in the plant that has not yet been polymerized into amorphous Si), even this portion not being abundant in the tissues, due to the silicon hyperaccumulation of this grass.

Soluble Si is represented by the most labile forms of Si, which are primarily composed of silicic acid [H_4_SiO_4_ or Si(OH)_4_, also called orthosilicic acid]^2^, whereas amorphous silica (SiO_2_) represents a non-soluble mineral with polar hydroxyl groups on its surface, which is able to adsorb other compounds depending on the degree of porosity, polymerization degree, and water content^22^. Several extractants of soluble Si have been studied by various researchers to evaluate Si availability in soil; however, none has been effective in all types of soils because the amounts of Si extracted vary greatly depending on the solution used in the extraction process^23,24^.

The soluble form of silica (H_4_SiO_4_) varies greatly between soils depending on their mineralogy^25,26^, where Si is predominantly found in sesquioxides, oxide hydroxides, and hydroxides of Fe and Al^15–17^, making primary and secondary minerals the main determinant of Si solubility in soil^15^, along with their texture^27–29^. Additionally, the high mobility of Si in most soils is suggested because leaching of Si from soils and its transport in streams to the sea are important components of the biogeochemical Si cycling, although such solubility decreases markedly with increasing pH, achieved, i.e., through liming application^15^.

Different extraction solutions have been used to evaluate Si availability in soils for plants. Acidic solutions have been considered more effective than neutral solutions, but in some cases, these solutions overestimate the levels of soluble Si^11,30,31^. Deionized water is used as a Si extractant from soil^28^ because it greatly dilutes the ionic strength of the soil solution and makes the amounts extracted different from those present in soil solution^15^. In addition, several others extractants have been used to estimate soluble Si in soil, such as: 0.5 mol L^−1^ acetic acid in the USA^23^ and Brazil^32^, 0.01 mol L^−1^ CaCl_2_ in Australia^33^ and Brazil^32^, phosphate buffer in Japan^34^, acetate buffer in Indonesia^35^ and Japan^34^, and sulphuric acid in Australia^36^. Despite this large number of extractants, the effectiveness of the extraction of soluble Si from soil depends not only on the extractant used but also on the extraction method itself as well as the combination of these two factors. Whereas 0.01 mol L^−1^ CaCl_2_, an unbuffered diluted salt, has an ionic strength similar to that of soil solution, thus avoiding dispersion and favoring Si extraction, Na-acetate and acetic acid provide an acid extraction, which results in the dissolution of amorphous Al and Fe oxides-hydroxides, amorphous and highly soluble crystalline aluminosilicates, with a Si release; this contributes to desorption of the adsorbed silicate anion from the acetate anion^15,37,38^, thus overestimating the available silicon content.

In Brazil, the world’s largest sugarcane producer, the evaluation of Si availability in soil has been performed using a CaCl_2_ (0.01 mol L^−1^) or acetic acid (0.5 mol L^−1^) extraction solution with the conventional method, which uses a horizontal shaker at 240 rpm for 1 h to stir the soil suspension/extractant^32^. However, alternative rapid methods can be used for Si extraction from soil. For example, extraction with a microwave oven has been used successfully for boron (B) extraction from soil^39,40^, and it has recently been used in the extraction of Si from leaf tissue samples^37^ and in silicate digestion of soil samples^41–44^. The use of a microwave oven as an extraction method of soluble Si from soil may be a rapid and effective method to quantify the Si availability in soils with different textures. The microwave oven method enables the analysis of a greater number of samples in a shorter time, with higher efficiency,because the duration of extraction is approximately six-fold less than in the conventional method with a horizontal shaker. The microwave oven method provides accurate and precise determination of the Si in soil and clay samples in most instances, even though it has not being considered a method that represents the solid-liquid exchanges in terrestrial soil environment. However, modifications to the procedure may be required for samples containing large quantities of primary Fe oxides and/or secondary Fe and Mn oxyhydroxides^45^, notably due to the effect of temperature on silicate solubility and microwave on colloidal dispersion.

Thus, the present study was conducted with the overall aim of assessing the effect of the extractable soil Si on Si uptake and stalk yield of sugarcane with the specific objectives of comparing two extraction methods (conventional and microwave oven) and five chemical Si extractants in soils with different textures.

## Materials and Methods

### Site description and soil sampling

Soil and plant samples were collected between March 2007 and December 2007 in Guaíra, state of São Paulo (SP), Brazil (20°19’ S; 48°18’ W; 520 m a.s.l.) and in Ariranha (SP) (20°23’ S; 49°25’ W; 590 m a.s.l.) for nine sampling areas, with no irrigation. According to Köeppen, the weather is Cwa, tropical humid, with rainfall in the summer and dry season during the winter. In the three areas of the Guaíra Sugar Mill, the soils are Oxisols, whereas in the six areas of the Colombo Sugar Mill, the soils are Ultisols^46^. To ensure that the study was representative of the sample areas, the nine areas were divided into four sampling points. Each sampling point was represented by a total area of 90 m^2^, i.e., six rows of sugarcane (10 m long and spaced 1.5 m apart). At each sampling point, five subsoil samples were collected (spaced 0.15 m in the sugarcane row) at depths from 0 to 0.20 and from 0.20 to 0.40 m. Subsoil samples were collected in an area of 60 m^2^ (four rows that were 10 m long and spaced 1.5 m apart) within the total area of each sampling point (90 m^2^).

### Sugarcane plant sampling and measurements

In the same area of soil sampling (60 m^2^), the stalk density was determined (stalk meter of row^−1^), and 20 stalks (stalk + leaves) were collected from each of the four sampling points. The stalks without leaves were weighed, and stalk yield per hectare was then estimated (Table 1). The stalks and leaves were transported to the laboratory, washed, shredded, dried in a forced-convection oven at 65 °C for 96 h and weighed. The dry matter and stalk density per meter were used to obtain the dry matter accumulation by the plants (in kg ha^−1^). The dried plant samples (stalk + leaves) were ground in a Wiley mill, and the Si concentration was determined after acid digestion in a closed vessel rotor using temperature-controlled microwave heating in a single step process followed by elemental determination in an inductively coupled plasma atomic emission spectroscopy (ICP-AES)^32,47^. The dry matter accumulation (kg ha^−1^) and Si concentration were used to obtain the amounts of Si accumulated by sugarcane plants (kg ha^−1^) (Table 1).

**Table 1:** Clay, Fe_2_O_3,_ Al_2_O_3,_ and SiO_2_ contents in the layer of 0.0 to 0.40 m depth, the amounts of Si absorbed by sugarcane plants, and sugarcane stalk yield in clayey, sandy-loam and sandy soils.

Site	Soil	Soil texture	Clay	Fe_2_O_3_	Al_2_O_3_	SiO_2_	Si absorbed	Stalk yield
			^_____________^ g kg^−1 _____________^				kg ha^−1^	Mg ha^−1^
1	Oxisol	Clayey	473	35.9	2.7	0.53	221	87
2	Oxisol	Clayey	470	37.4	2.3	0.42	204	85
3	Oxisol	Clayey	502	34.0	2.1	0.33	167	73
4	Ultisol	Sandy-loam	226	10.6	0.9	0.37	338	123
5	Ultisol	Sandy-loam	218	12.3	0.8	0.38	299	131
6	Ultisol	Sandy-loam	193	12.9	0.9	0.30	283	108
7	Ultisol	Sandy	112	10.0	0.5	0.26	265	109
8	Ultisol	Sandy	109	9.1	0.6	0.31	215	102
9	Ultisol	Sandy	99	9.3	0.6	0.23	288	125

Mean values of 24 replicates.

### Soil analysis, experimental design and treatments

The soil samples were air dried, sieved with a 2-mm mesh and analyzed for the determination of clay, Fe oxide (Fe_2_O_3_), Al oxide (Al_2_O_3_), and Si oxide (SiO_2_) contents, according to Brazilian Agricultural Research Corporation methods^48^ based on Mehra and Jackson^49^ and McKeague and Day^50^ (Table 1). For the Fe_2_O_3_ and Al_2_O_3_ determination, we performed the dithionite citrate bicarbonate (DCB) extraction based on Mehra and Jackson^49^. The sample is heated in a complexing DCB buffered solution, to which powdered sodium dithionite is added as a reducing agent. Iron and aluminum were determined in the extract by AAS (atomic absorption spectroscopy).

Based on the clay contents, samples collected in the nine areas were divided according to soil textures as follows: clayey, sandy-loam and sandy soils. The soil in the three areas containing Oxisol had a clayey texture. The soil in three of the six areas containing Ultisol had a sandy-loam texture, and the soil in the other three areas containing Ultisol had a sandy texture. Qualitative and quantitative soil mineralogy, as well as weathering stage, SiO_2_ contents and pH values, around both municipalities Guaíra and Ariranha, can be found in Alves and Lavorenti^51^, Soares *et al*.^52^, Alves and Omotoso^53^, and Camargo and Marques Júnior^54,55^. The soil minerals consist essentially of kaolinite, hematite, goethite, gibbsite and quartz.

The experiment was conducted in a completely randomized design in a 2 × 5 factorial scheme with 24 replications. The treatments consisted of the conventional and microwave oven extraction methods, and the extractants were calcium chloride (0.01 mol L^−1^), deionized water, potassium chloride (0.01 mol L^−1^), Na-acetate buffer (pH 4.0) and acetic acid (0.5 mol L^−1^).

### Extraction and determination of Si in the soil

The preparation of soil samples for the extraction of available Si was performed according to Korndörfer *et al*.^32^, adapted from Haysom and Chapman^56^, Imaizumi and Yoshida^57^, Fox *et al*.^58^, Elawad *et al*.^59^, found in Haynes^15^, and presented in Liang *et al*.^6^. The conventional extraction method^32^ was performed by placing 10 g of soil in a 150-mL plastic bottle followed by the addition of 100 mL of each of the extraction solutions (extractants: CaCl_2_, deionized water, KCl, Na-acetate buffer (pH 4.0), and acetic acid). The plastic bottles were agitated in a horizontal shaker at 240 rpm for 1 h. The material was then allowed to stand for 15 minutes, and the suspension was filtered using quantitative filter paper. The solution was then allowed to stand overnight (period longer than 12 h), and this solution was used to determine the Si content according to the procedures described in Korndörfer *et al*.^32^.

For the Si extraction using a microwave oven, a 35 L microwave equipped with a polypropylene support for 14 plastic bottles of 100 mL with caps was used. The plastic bottle caps were pierced in the center to allow the release of vapors generated at the time of boiling, which was similar to the method adopted in the procedure for the extraction of B from the soil^39^. Before starting the extraction process, the microwave oven was calibrated to perform the power and time of heating during the extraction process, following the methodology of Chaves *et al*.^60^. Initially, 1,000 g of distilled water at ambient temperature (23 ± 2 °C) was added into a beaker, and the initial temperature (Ti) was recorded. The beaker was covered, placed in the microwave oven, and heated for 2 minutes at maximum power (100%). At the end of heating, the water in the beaker was stirred vigorously, and the temperature was recorded (Tf). This same procedure was repeated at 80%, 60%, 40%, and 20% of maximum power, always with distilled water at ambient temperature. The power of the equipment was calculated using equation (1):

1 $${\rm{P}}(\mathrm{watts})=({\rm{K}}\,{\rm{Cp}}\,m{\rm{\Delta }}T)/{\rm{t}}$$

where P is the apparent power absorbed by the sample, in watts (W) or joules sec^−1^, K is the conversion factor of thermochemical calories to watts (4.184 joule cal^−1^), Cp is the capacity of heating or specific heat (1.00 g cal^−1^ °C^−1^), m is the mass of the water sample in grams (g), ∆T is the difference between the final and initial temperatures (°C), and t is time in seconds (s).

Before making the Si extraction using the microwave oven method, we performed preliminary tests using the five extractants (CaCl_2_, deionized water, KCl, Na-acetate buffer (pH 4.0), and acetic acid) and a standard soil sample, which contained 10 mg dm^−3^ of soluble Si as determined using the conventional method and pattern extractant (CaCl_2_ 0.01 mol L^−1^)^32,56^. These tests determined the best heating programs during the extraction process of Si. The best results were obtained with the heating program at a power of 663 watts for 4 minutes followed by a power of 383 watts for five minutes. This appliance power was calibrated in the presence of the support and 14 bottles of soil samples. However, if fewer than 14 soil samples were used, additional plastic bottles containing extraction solution without soil samples were used to maintain the proper power calibration.

After preliminary tests, the Si extraction from soil was conducted by placing 5 g of soil and 50 mL of the extractant to be analyzed in each plastic bottle. The plastic bottles were then sealed with pierced caps and placed in the center of the microwave oven. The extraction was performed at a power of 663 watts for 4 minutes followed by a power of 383 watts for five minutes. The support with the samples was removed from the microwave oven, and the samples remained at rest for 15 minutes. The suspension was then filtered with quantitative filter paper, and the solution obtained was used to determine the Si content of this solution according to the procedures described by Korndörfer *et al*.^32^. For this, a 10-mL aliquot of the extracts filtered from each sample was pipetted and placed in a 50-mL beaker. 0, 0.4, 1.0, and 2.0 mg L^−1^ of Si were used as standards to obtain the calibration curve. Zero, 2, 5, and 10 mL of the 20 mg L^−1^ Si standard solution were pipetted in a 100-mL volumetric flask and the volume completed with deionized water. A 10-mL aliquot was then removed from each standard and placed in a 50-mL beaker. One milliliter of sulfo-molybdic solution was added to the volumes of both standards and samples. After 10 minutes, 2 mL of the 200 mg L^−1^ tartaric acid solution was added followed by 10 mL of ascorbic acid solution after a further 5 minutes. Finally, after a 1 hour-rest, a spectrophotometer reading was taken at a wavelength of 660 nm.

### Statistical data analysis

All data were initially tested for normality with the Shapiro-Wilk test^61^ and the results showed that all data were distributed normally. The soil Si concentration results were separated by soil texture classes (clayey, sandy-loam, and sandy textures), and they were submitted to ANOVA separately. The means of Si concentrations in the soil were compared using the LSD test at *P* ≤ 0.05. The Si extracted using the different methods and extractants from the three textural classes were correlated using the Pearson (r) test at *P* ≤ 0.05 with the contents of clay, Fe_2_O_3_, Al_2_O_3_, and SiO_2_, the amounts of Si accumulated in the aboveground parts of sugarcane, and the sugarcane stalk yields. The statistical analyses were performed using the SAS^62^ statistical software package.

## Results

**Figure 1:**
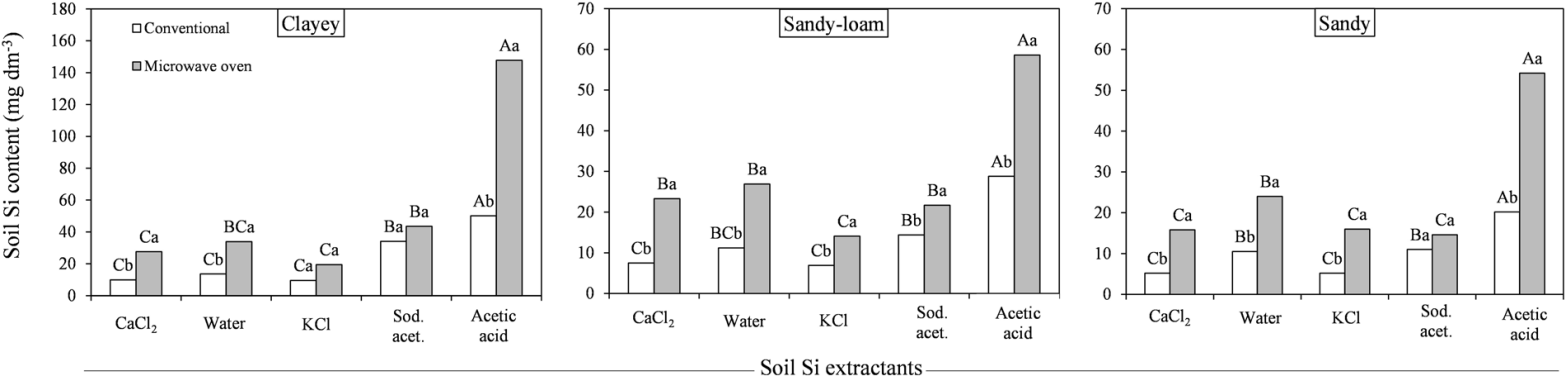
Silicon contents in three soil texture classes using conventional (□) and microwave oven (■) methods with the CaCl_2_, deionized water, KCl, Na-acetate buffer (pH 4.0) (Sod. acet.) and acetic acid extractants. Means followed by the same uppercase letter within the same method or lowercase within the same extractant for each soil texture are not significantly different (P < 0.05, LSD test).

The estimates of soluble soil Si were affected by the method × extractant interaction (*P* ≤ 0.01). In all the texture classes, the microwave oven method extracted more Si from soil than the conventional method, except when the KCl and Na-acetate buffer (pH 4.0) extractants were used, especially in clayey soils (Fig. 1).

In both extraction methods and in all soil textures, a higher Si content was obtained when the extraction was performed with acetic acid, especially in clayey soils (Fig. 1). Acetic acid extracted more than two-fold the amount of Si when the extraction was performed with the microwave oven method (*P* ≤ 0.01). Silicon extraction using the conventional method with the Na-acetate buffer (pH 4.0) and deionized water obtained intermediate Si contents from soil, especially from the sandy-loam and sandy soils. In the clayey soils, the conventional method with deionized water extracted similar Si contents to those extracted with CaCl_2_ and KCl extractants (*P* ≤ 0.01).

The soil Si contents were similar in clayey soils when the extraction was performed with the microwave oven and the deionized water and Na-acetate buffer (pH 4.0) extractants (*P* ≤ 0.01), but the soil Si contents resulting from the Na-acetate buffer (pH 4.0) extractant were higher than those obtained with the CaCl_2_ and KCl extractants (Fig. 1). In the sandy-loam soils, the CaCl_2_, deionized water and Na-acetate buffer (pH 4.0) extractants used with the microwave oven method extracted similar Si contents from soil, but deionized water extracted more Si than all of these extractants in sandy soils. Regardless of the method and/or extractant used, the highest soluble Si contents were obtained from clayey soils followed by sandy-loam and sandy soils.

**Figure 2:**
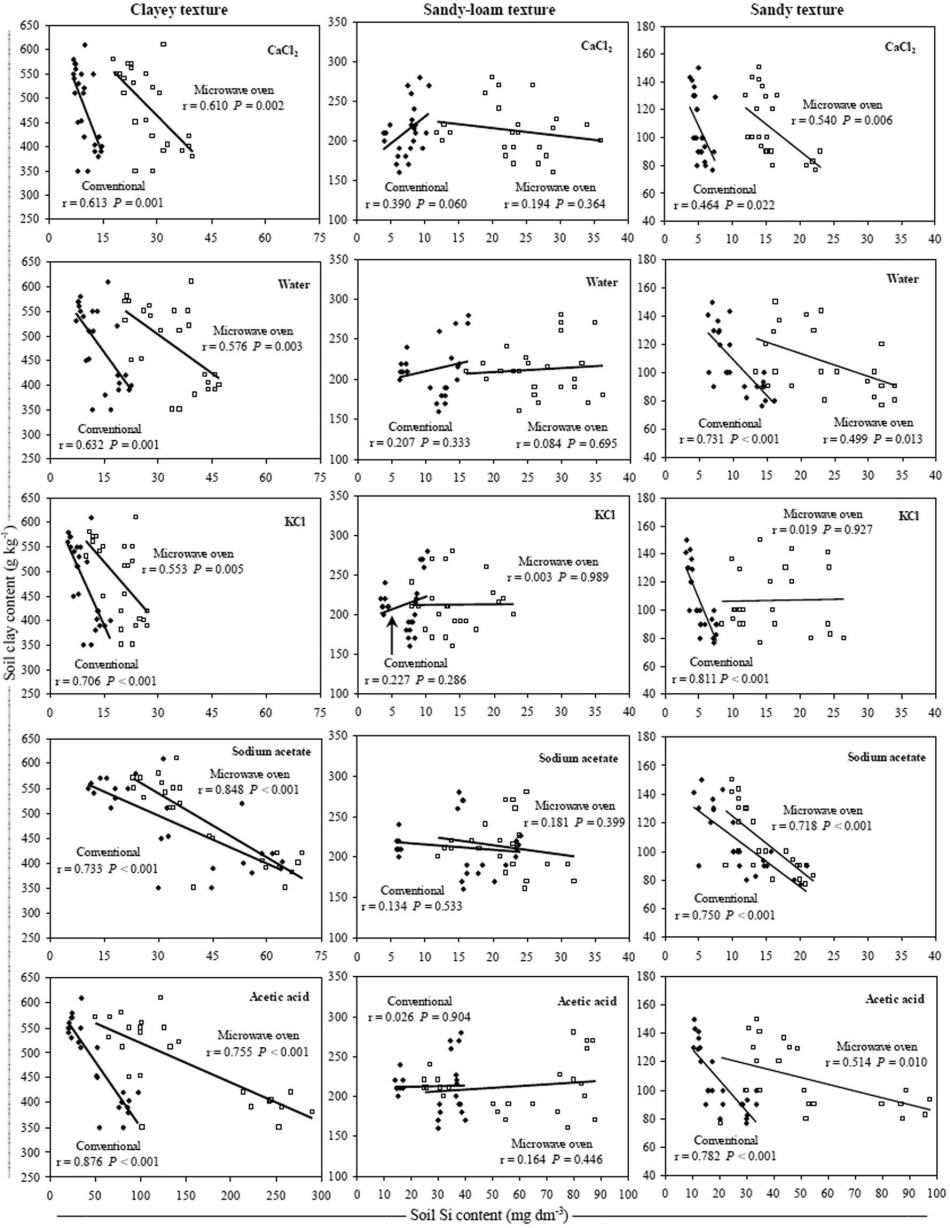
Relationship between clay and Si contents in three soil texture classes using conventional (◆) and microwave oven (□) methods with CaCl_2_, deionized water, KCl, Na-acetate buffer (pH 4.0) and acetic acid extractants. Note: when comparing soil textures it is important to take in account the differences between the axes scales.

**Figure 3:**
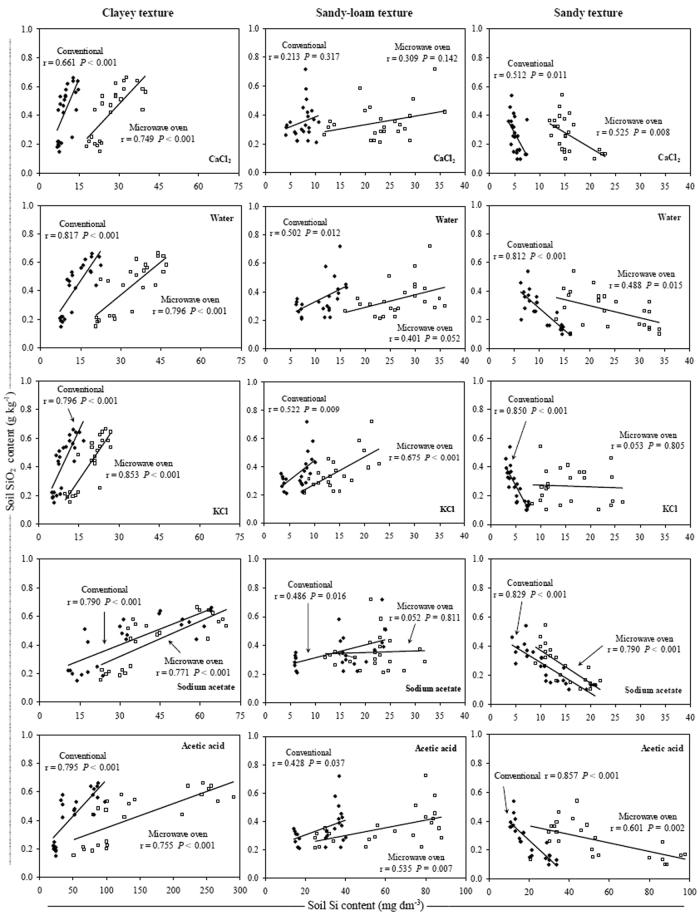
Relationship between SiO_2_ and Si content in three soil texture classes using conventional (◆) and microwave oven (□) methods with CaCl_2_, deionized water, KCl, Na-acetate buffer (pH 4.0) and acetic acid extractants. Note: when comparing soil textures it is important to take in account the differences between the axes scales.

The soluble Si contents were negatively correlated with the soil clay content only in clayey and sandy soils, and with SiO_2_ content in sandy soils (Figs 2 and 3). In clayey soils, the highest correlation between the soluble Si and clay contents occurred with extraction by acetic acid and Na-acetate buffer (pH 4.0) in both extraction methods (Fig. 2). However, in sandy soils the clay contents were better correlated with the Si contents when the KCl extractant was used with the conventional method, while the better correlation among the soluble Si and SiO_2_ contents, occurred with extraction by acetic acid in conventional method (Figs 2 and 3). In clayey and sandy-loam soils, the soluble Si content was positively correlated with the soil SiO_2_ content, and the higher correlation coefficient values between these variables were obtained with the use of KCl extractant in the microwave oven method (Fig. 3).

**Figure 4:**
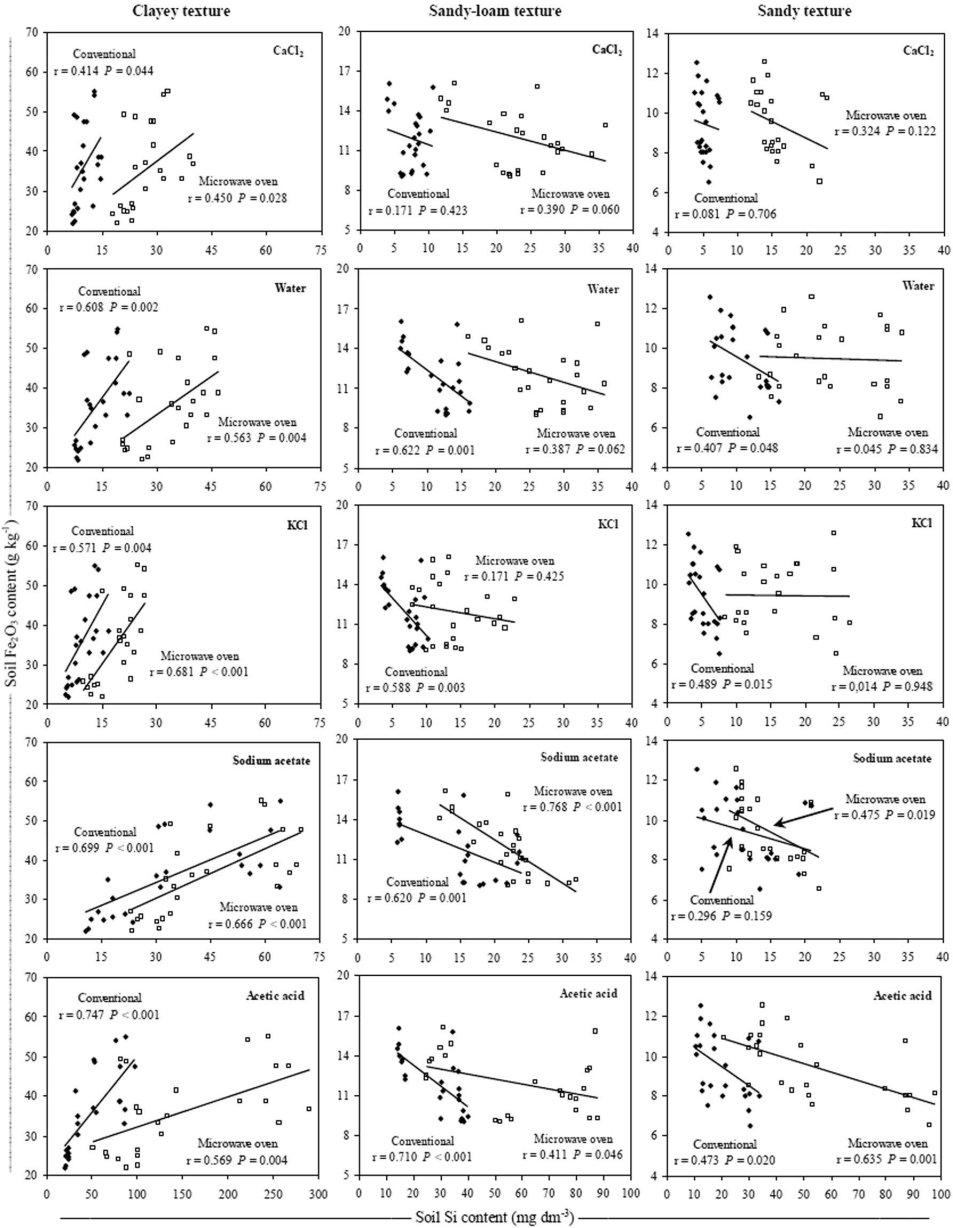
Relationship between Fe_2_O_3_ and Si content in three soil texture classes using conventional (◆) and microwave oven (□) methods with CaCl_2_, deionized water, KCl, Na-acetate buffer (pH 4.0) and acetic acid extractants. Note: when comparing soil textures it is important to take in account the differences between the axes scales.

**Figure 5:**
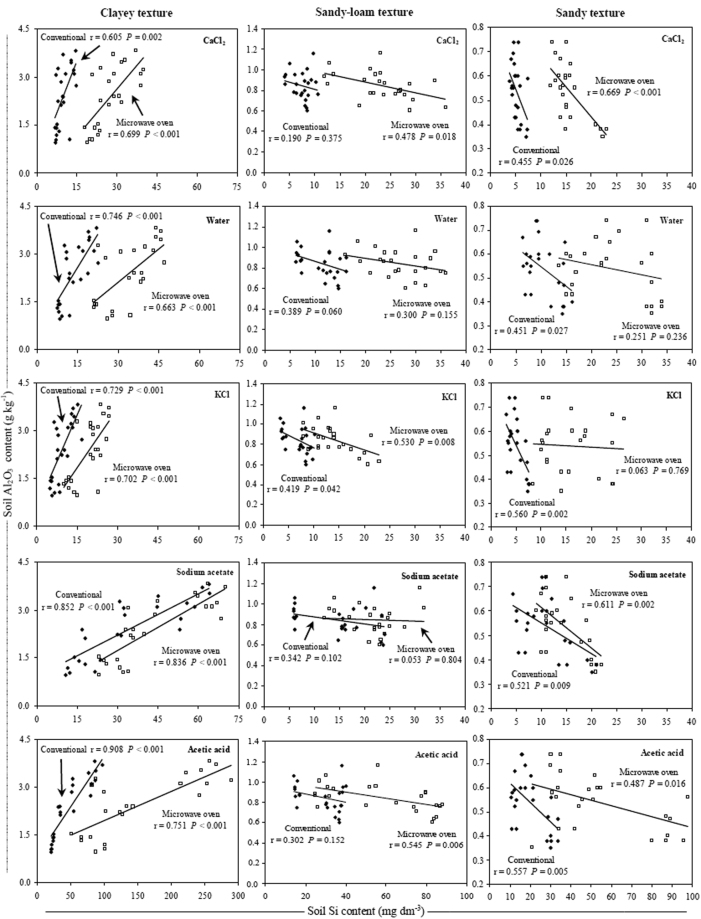
Relationship between Al_2_O_3_ and Si content in three soil texture classes using conventional (◆) and microwave oven (□) methods with CaCl_2_, deionized water, KCl, Na-acetate buffer (pH 4.0) and acetic acid extractants. Note: when comparing soil textures it is important to take in account the differences between the axes scales.

Regardless of the method and extractant used, the soil Si contents were positively correlated with the soil Fe_2_O_3_ and Al_2_O_3_ contents in the clayey soils (Figs 4 and 5). When soil Si content was correlated with Fe_2_O_3_ and Al_2_O_3_ contents in the sandy-loam and sandy soils, the correlation was negative or there was no correlation between these variables for most extractants and/or methods.

**Figure 6:**
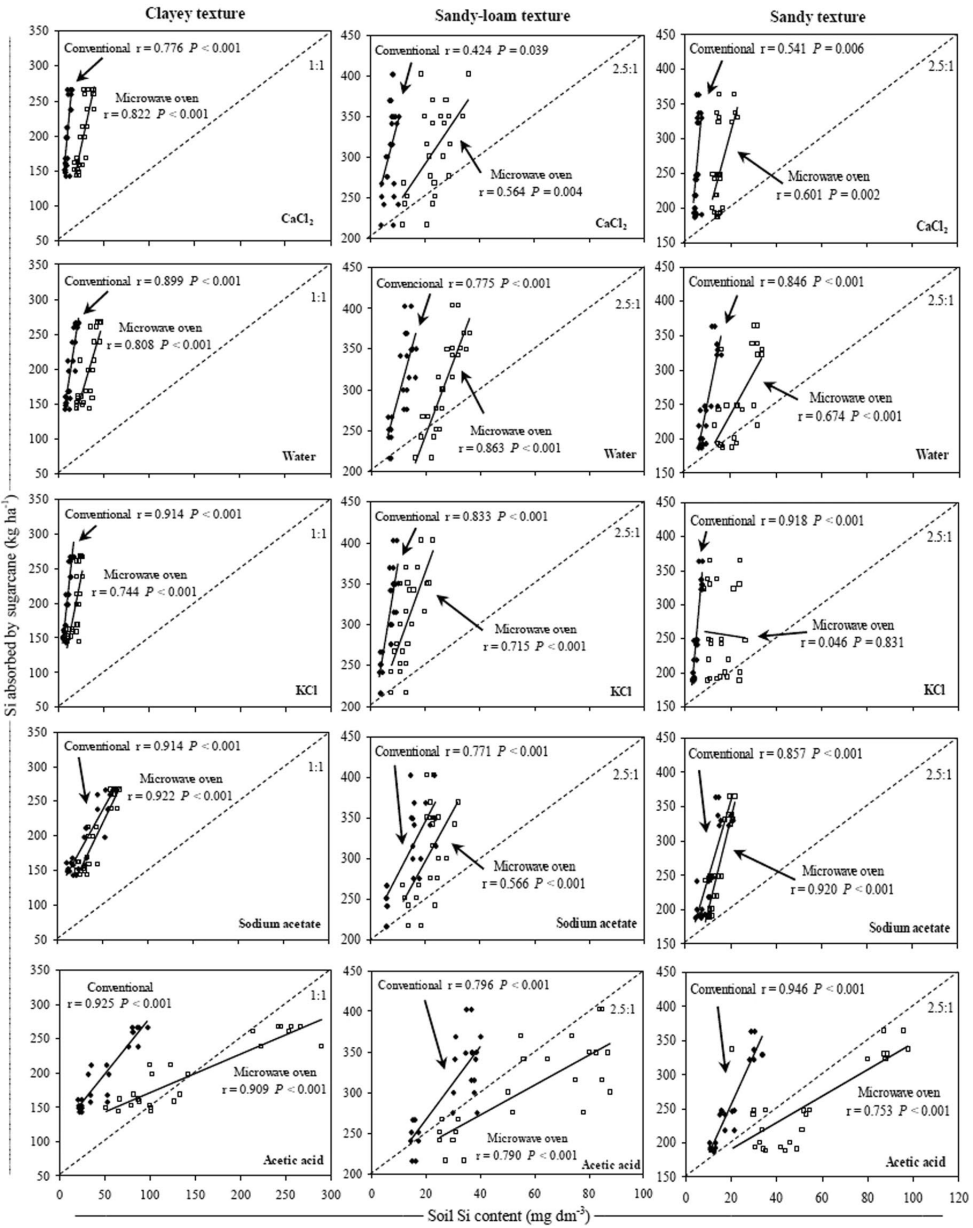
Relationship between Si absorbed by sugarcane and Si content in three soil texture classes using conventional (◆) and microwave oven (□) methods with CaCl_2_, deionized water, KCl, Na-acetate buffer (pH 4.0) and acetic acid extractants. Dashed line indicates the relationship of increase between the variables. Note: when comparing soil textures it is important to take in account the differences between the axes scales.

For all extraction methods and in most soil textural classes, positive and significant correlations were found with soil Si contents and the amounts of Si absorbed by sugarcane (Fig. 6). The KCl extractant with the conventional method was the extraction method that showed the highest correlation coefficient values between soil Si content and the amount of Si absorbed by sugarcane in the three soil textural classes. Extraction using Na-acetate buffer (pH 4.0) with both methods in clayey soils and with the microwave oven method in sandy soils also resulted in high correlation coefficient values between the soil soluble Si (available content) and the Si absorbed by sugarcane (Fig. 6).

The acetic acid extractant with both methods in clay soils and with the conventional method in sandy soils resulted in high correlation coefficient values between the soil Si content and the Si absorbed by sugarcane, but extraction with acetic acid using the microwave oven method overestimated the soil Si availability (Fig. 6). In sandy-loam soils, extraction using deionized water with the microwave oven method resulted in correlation coefficient values greater than extraction using KCl with the conventional method, but both forms of extraction resulted in the best estimates of soluble soil Si contents. In all soil textural classes, extraction using CaCl_2_ with both methods resulted in lower correlation coefficient values between the Si absorbed by sugarcane and the soil Si content (Fig. 6).

**Figure 7:**
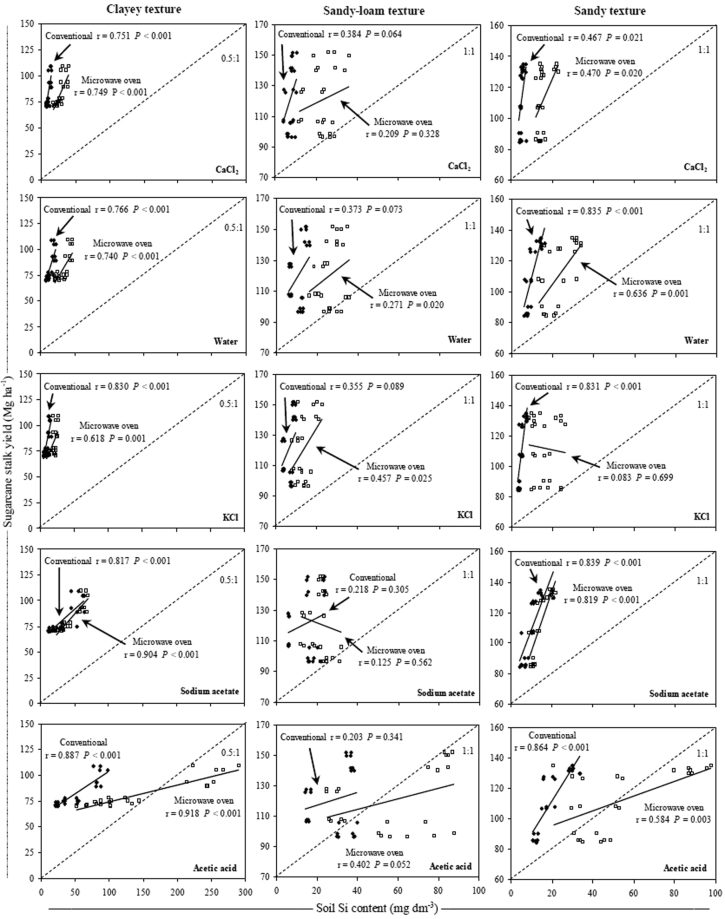
Relationship between sugarcane stalk yield and Si content in three soil texture classes using conventional (◆) and microwave oven (□) methods with CaCl_2_, deionized water, KCl, Na-acetate buffer (pH 4.0) and acetic acid extractants. Dashed line indicates the relationship of increase between the variables. Note: when comparing soil textures it is important to take in account the differences between the axes scales.

There was no significant correlation between sugarcane stalk yield and the soil Si content in the sandy-loam soils (Fig. 7). There was also no correlation between sugarcane stalk yield and the amounts of Si absorbed by sugarcane in these soils (Table 2). In clayey soils, the extraction using acetic acid and Na-acetate buffer (pH 4.0) with the microwave oven method resulted in the highest correlation coefficients of soil Si content with sugarcane stalk yield, but the best correlations in sandy soils were obtained with the use of these extractants in the conventional method (Fig. 7).

**Table 2:** Pearson correlation coefficients (r) and t probability (P) between the amounts of Si absorbed by sugarcane and sugarcane stalk yield in soils with clayey, sandy-loam and sandy textures.

Characteristic		Si absorbed by sugarcane		
		Clayey texture	Sandy-loam texture	Sandy texture
Sugarcane stalk yield	r	0.851	0.395	0.891
	*P*	<0.001	0.056	<0.001

In the extraction using acetic acid with the microwave oven method, the Si content increased by a larger proportion than sugarcane stalk yield (Fig. 7). In general, extraction using the microwave oven resulted in lower correlations of sugarcane stalk yield with the soil Si content in the sandy soils, which did not occur in the extraction with the conventional method. In the clayey and sandy soils, the extraction using KCl resulted in high correlation coefficient values between sugarcane stalk yield and soil Si content, especially using the conventional method. However, in both clayey and sandy soils, the lowest correlation coefficient values between sugarcane stalk yield and soil Si content were obtained when using CaCl_2_ and deionized water, especially when these extractants were used with the microwave oven method.

## Discussion

The soil Si extraction was higher with the use of the microwave oven method (Fig. 1) due to the effect of temperature on Si solubilization because the increase in temperature reduces silica adsorption in the solid phase of soil and increases the dissolution of silica in soil^25^. Xu *et al*.^31^ also observed that the increase in temperature caused by changes in the method of soil Si extraction results in higher estimates of soluble soil Si. The acetic acid extractant resulted in higher extracted soil Si contents, especially in clayey soils (Fig. 1 and Table 1), which had higher contents of Fe_2_O_3_ and Al_2_O_3_ (i.e., where the soluble Si preferentially binds in these sesquioxides)^16–18^. Thus, acetic acid may have overestimated the Si availability in the soil because this extractant can solubilize low-solubility forms of Si, which are often not available to plants^19,24^. Our results showed that an increase in temperature during the extraction process in conjunction with the capacity of acetic acid to extract the non-soluble forms of Si in soil (non-phyto available), resulted in an even greater overestimation of current Si availability in the soil (Fig. 1).

In the sandy-loam and sandy soils, the soil Si contents were intermediate when deionized water and Na-acetate buffer (pH 4.0) extractants were used with the conventional method (Fig. 1). In clayey soils, using deionized water with the conventional method had the same Si extraction capacity as using this method with the CaCl_2_ and KCl extractants, which were less effective in the Si extraction. In the clayey soils, the extraction with the microwave oven method using the Na-acetate buffer (pH 4.0) and deionized water had the same capacity to extract Si from soil, but these extractants were more effective than the CaCl_2_ and KCl extractants (Fig. 1). In the sandy-loam soils, the capacity of Si extraction using the microwave oven method was similar among the CaCl_2_, deionized water and Na-acetate buffer (pH 4.0) extractants, but deionized water extracted more Si from sandy soils than the other extractants. In general, higher soil soluble Si contents were obtained from the clayey soils followed by sandy-loam and sandy soils (Fig. 1), a result that was also found by Freitas *et al*.^29^, maybe due to an easier Si solubilization from smaller particles (clay).

The negative correlation between the soil Si content and soil clay content indicated that not all soils with higher clay contents had lower soluble Si contents (Fig. 2). The soluble Si contents should also be associated with the type of soil and not just to the soil clay content because there are mineralogical differences among highly-weathered soils, such as Oxisols and Ultisols in the case of this study, mainly related to the quantity of oxides, oxi-hydroxides and silicated minerals in their surface horizons^51–55^, that probably result in available Si contents not simply explained by soil clay content^10^. A previous study using four types of soil (Typic Udorthent, Ultisol, Oxisol and Udox) demonstrated a negative correlation between the soil clay and soluble Si contents only in the Ultisol^29^, which agreed with the results of this study.

When acetic acid and Na-acetate buffer (pH 4.0) were used for extraction in the clayey soils, a higher correlation between the soil Si content and the soil clay content indicated that these extractants were more effective in extracting the soluble Si forms present in these Oxisols, which have higher Fe_2_O_3_, Al_2_O_3_ and SiO_2_ contents (Fig. 2 and Table 1). In highly-weathered soils from tropical regions, which are more prone to advanced stages of weathering and desilication, Si is significantly found in these sesquioxides^16–18^.

In sandy soils, the best correlation coefficients of soil soluble Si with soil clay content occurred with the use of KCl in the conventional method (Fig. 2) because the sandy and sandy-loam soils are all Ultisols, which are less weathered and contain a higher proportion of soluble Si in the soil^10^. Therefore, the extractant with the best correlation between the soil Si content and the soil clay content was not the extractant with a higher capacity to extract Si from soil (Figs 1 and 2). This result suggested that in less weathered soils with low Fe_2_O_3_ and Al_2_O_3_ contents and with a greater presence of minerals (1:1), such as kaolinite, the Si release to soil solution is higher^10^ due to the higher presence of silicated minerals in comparison to highly-weathered soils. In these soils, the Si was not strongly adsorbed to Fe_2_O_3_ and Al_2_O_3_, such as occurs in clayey soil and more weathered soils (Figs 1, 2, 4, 5, and Table 1).

In the clayey soils, there was a positive correlation between soil soluble Si and Fe_2_O_3_ and Al_2_O_3_ contents, but when there was a correlation between these variables in the sandy-loam and sandy soils, it was negative (Figs 4 and 5). This result shows that, generally, in most highly-weathered tropical soils, Fe_2_O_3_ and Al_2_O_3_ strongly bind silicates^19^ because soluble Si preferentially binds these sesquioxides^16–18^. Because the Fe_2_O_3_ and Al_2_O_3_ contents are higher in clayey soils (Table 1), any method and/or extractant used to quantify the Si availability results in higher Si contents than those obtained in soils with lower clay content (Fig. 1). In the sandy-loam and sandy soils, however, none of the methods, including extractants, resulted in a positive correlation between these variables because the soluble Si was not adsorbed by Fe_2_O_3_ and Al_2_O_3_ (Figs 4 and 5).

There was a positive and significant correlation between the soil Si content and the amounts of Si accumulated by sugarcane in all soil textural classes (Fig. 6). Despite an underestimation of the Si availability in soil, only the KCl extractant with the conventional method showed a high correlation coefficient of Si absorbed with soil soluble Si content in all soil textural classes with values between 0.833 and 0.918 (Fig. 6). These results indicate that this method has less variation between soils with different textural classes when estimating soluble Si. The extractants solubilize Si in different components of the soil matrix, and the saline solutions, such as KCl, primarily extract the Si content available in the soil solution^33^. Nevertheless, this extractant best estimated the Si availability in clayey soils in which most of the Si is adsorbed by Fe_2_O_3_ and Al_2_O_3_^18,19^, demonstrating that this extractant can solubilize part of the labile Si adsorbed by Fe_2_O_3_ and Al_2_O_3_ in addition to measuring the readily available Si (Fig. 6).

The Na-acetate buffer (pH 4.0) was an effective extractant to evaluate the Si availability in clayey and sandy soils, except when it was used with the conventional method in the sandy soils (Fig. 6). According to Liang *et al*.^63^, the use of Na-acetate overestimates the amount of Si available to plants in calcareous soils with high soluble Si content because the crops continue to respond to the Si supply under these conditions. However, in the tropical soil conditions in which the present study was conducted, the use of Na-acetate buffer (pH 4.0) did not overestimate the Si availability to plants because the amounts of Si absorbed by sugarcane increased more than in direct proportion to the extractable soil soluble Si (Fig. 6).

Although the acetic acid extraction exhibited a high correlation of soil Si content with the amounts of Si absorbed by sugarcane in all textural classes, the use of this extractant with the microwave oven method overestimated the Si availability for this crop because the soil Si content increased more than in direct proportion to the amounts of Si absorbed by sugarcane (Fig. 6). This result occurred because acetic acid can extract low-solubility Si forms^19^ and Si from sources applied to the soil in the polymerized form (polysilicic acid), which are generally not soluble by other extractants, such as CaCl_2_^11^. According to Pereira *et al*.^64^, acetic acid generally overestimates the Si content in soils, especially in limed soils and those that receive applications of sources rich in aluminosilicates, such as blast furnace slag. However, in the present study, acetic acid with the conventional method did not overestimate the Si availability in the soil and provided high correlation coefficients between the soluble Si content in soil and the amounts of Si absorbed by sugarcane, especially in clayey and sandy soils (Fig. 6).

In the sandy-loam soils, the best estimate of Si availability in the soil was obtained by extraction using deionized water with the microwave oven method (Fig. 6). Pereira *et al*.^65^, in a study on different Si sources, observed that water extracts less Si than acetic acid but that water is a safer extractant because it does not overestimate the soluble Si from sources of Si applied to soil. Although deionized water has been effective in estimating the Si availability in sandy-loam soils, this extractant inconveniently disperses the clay particles during the extraction process, thus requiring a longer time to decant before filtering^66^ or a higher centrifugation speed to separate the solution (water + Si) of the clay prior to the Si determination^28^.

Regardless of the method used, CaCl_2_ was not effective for evaluating the Si availability in soils with sugarcane due to the low correlation between the amount of Si absorbed by sugarcane and the soil Si content (Fig. 6). However, CaCl_2_ has been the primary Si extractant used in Brazil^32^. According to Lima Filho^2^, CaCl_2_ extracts small amounts of Si from soil but can better estimate the available Si in the soil solution, which contradicts the results of the present study. According to Haynes *et al*.^67^, saline solutions, such as diluted CaCl_2_, primarily measures the Si content available in the soil solution. However, CaCl_2_ was not more effective than the other extractants for evaluating Si availability even in sandy soil in which most of the Si is not adsorbed by Fe_2_O_3_ and Al_2_O_3_ (Figs. 4, 5, 6, and Table 1). The low correlation coefficient values obtained with CaCl_2_ in the soils with high clay content also indicated the ineffectiveness of this extractant in solubilizing the Si that is adsorbed by Fe_2_O_3_ and Al_2_O_3_^18,19^ (Figs 1, 4, 5, 6).

In the sandy-loam soils, there was no significant correlation between sugarcane stalk yield and the soluble Si content in soil because the sugarcane stalk yield was not correlated with the amount of Si absorbed by sugarcane in these soils (Fig. 7 and Table 2). These results indicated that greater Si absorption by sugarcane does not always indicate a higher stalk yield, demonstrating that the correlation between sugarcane stalk yield and the soil Si content is not a good indicator of Si availability for sugarcane crops. Moreover, sandy-loam soils naturally present a lower cation exchange capacity (CEC) in comparison to clayey soils, which can significantly affect the stalk yield and Si uptake by sugarcane^68^.

The acetic acid and Na-acetate buffer (pH 4.0) extractants had the best correlations between Si available in the soil and sugarcane stalk yield in clayey and sandy soils using the microwave oven and conventional methods, respectively (Fig. 7). For rice crop (*Oryza sativa* L.), a high correlation of plant dry matter yield with the Si availability in the soil has been found when acetic acid is used as the extractant^66^. Rice grain yield is also correlated with the soil Si content when Si is extracted either with acetic acid or deionized water^69^.

When Si extraction was performed with acetic acid using the microwave oven method, the soil Si content increased to a greater degree than sugarcane stalk yield (Fig. 7). This result indicates that acetic acid overestimates the Si availability for sugarcane with this extraction method due to the increase in temperature during the extraction process, which increases the solubilization of the non-soluble forms of Si in the soil (Figs 1 and 7). Moreover, the contents of soluble Si obtained with acetic acid are greatly influenced by variations in the soil pH, which is highly dependent on the acidity correction and can lead to an overestimation of soluble Si available to plants in soils, as is the case of Brazilian soils^10^.

The extraction using KCl with the conventional method in clayey and sandy soils resulted in the highest correlation between sugarcane stalk yield and soil Si contents, whereas smaller correlations occurred with the use of CaCl_2_ and deionized water, especially when these extractants were used with the microwave oven method. Our results showed that CaCl_2_, which is the primary soil Si extractant used in Brazil^32^, Australia^67^ and South Africa^70^, is not effective in evaluating the Si available to sugarcane regardless of the extraction method.

## Conclusion

No single method and/or extractant adequately estimated the Si availability in the soil for sugarcane grown in soils with different textures. The conventional extraction with KCl was no more effective than other methods in evaluating Si availability; however, conventional extraction showed less variation in estimating soluble Si between soils with different textural classes. The microwave oven method estimated the Si availability in the soil with effectiveness similar to or even higher than the conventional method depending on the soil texture and extractant used. In the clayey and sandy soils, the Na-acetate buffer (pH 4.0) and acetic acid with both extraction methods were effective in evaluating the Si availability in soil, but extraction using acetic acid with the microwave oven method overestimated the Si availability in soil. In the sandy-loam soil, extraction using deionized water with the microwave oven method was more effective in estimating the Si availability in soil than the other extraction methods.
